# BAG6 prevents the aggregation of neurodegeneration-associated fragments of TDP43

**DOI:** 10.1016/j.isci.2022.104273

**Published:** 2022-04-20

**Authors:** Yasar Arfat T. Kasu, Akshaya Arva, Jess Johnson, Christin Sajan, Jasmin Manzano, Andrew Hennes, Jacy Haynes, Christopher S. Brower

**Affiliations:** 1 Department of Biology, Texas Woman’s University, P.O. Box 425799, Denton, TX 76204, USA

**Keywords:** Biological sciences, Molecular biology, Neuroscience, Molecular neuroscience, Cell biology

## Abstract

Neurodegeneration is associated with the aggregation of proteins bearing solvent-exposed hydrophobicity as a result of their misfolding and/or proteolytic cleavage. An understanding of the cellular protein quality control mechanisms which prevent protein aggregation is fundamental to understanding the etiology of neurodegeneration. By examining the metabolism of disease-linked C-terminal fragments of the TAR DNA-binding protein 43 (TDP43), we found that the Bcl-2 associated athanogene 6 (BAG6) functions as a sensor of proteolytic fragments bearing exposed hydrophobicity and prevents their intracellular aggregation. In addition, BAG6 facilitates the ubiquitylation of TDP43 fragments by recruiting the Ub-ligase, Ring finger protein 126 (RNF126). Authenticating its role in preventing aggregation, we found that TDP43 fragments form intracellular aggregates in the absence of BAG6. Finally, we found that BAG6 could interact with and solubilize additional neurodegeneration-associated proteolytic fragments. Therefore, BAG6 plays a general role in preventing intracellular aggregation associated with neurodegeneration.

## Highlights

• Proteolytic cleavage generates protein fragments bearing exposed hydrophobicity
• BAG6 maintains the solubility and directs the degradation of protein fragments
• BAG6 prevents intracellular aggregation associated with neurodegeneration

## Graphical abstract

**Figure:**
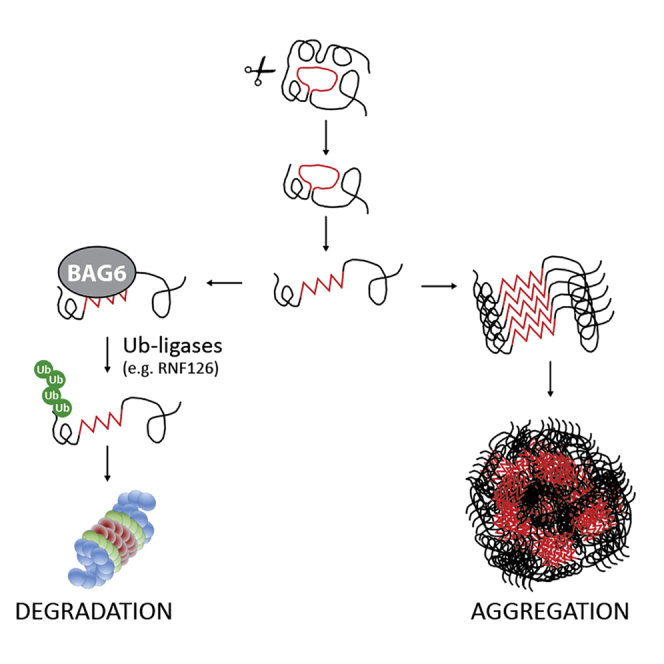

## Introduction

Neurodegenerative diseases such as Alzheimer’s disease (AD), Parkinson’s disease, Huntington’s disease, amyotrophic lateral sclerosis (ALS), and frontotemporal degeneration (FTD) are associated with the accumulation and aggregation of specific neuronal proteins. As a consequence of their misfolding, normally obscured hydrophobic portions of proteins become solvent-exposed, leading to their self-association. Proteolytic cleavages generate polypeptide fragments which can also self-interact to form a spectrum of species ranging from soluble oligomers to large insoluble proteinaceous aggregates (Ross and Poirier, 2004; Ciechanover and Kwon, 2017; Mathieu et al., 2020). Protein aggregation and its associated toxicity is typically mitigated through various protein quality control (PQC) mechanisms that prevent aggregation by facilitating protein removal through the autophagy-lysosomal system or the ubiquitin proteasome system (UPS) (Rubinsztein, 2006; Douglas and Dillin, 2010; Ciechanover and Kwon, 2015; Dubnikov et al., 2017). Indeed a number of aging-related neurodegenerative disorders have been associated with defects in autophagy or UPS activity (Komatsu et al., 2006; Kourtis and Tavernarakis, 2011; Harris and Rubinsztein, 2012; Tashiro et al., 2012).

The Bcl-2 associated athanogene 6 (BAG6) is a molecular “holdase” chaperone that binds to “client” proteins bearing solvent-exposed hydrophobicity and determines their fate through interactions with various downstream effectors (Gottesman et al., 1997; Kawahara et al., 2013; Hessa et al., 2011; Shao et al., 2017; Ganji et al., 2018; Kamikubo et al., 2019). BAG6 assembles with the transmembrane recognition complex 35 (TRC35) and the ubiquitin-like protein 4A (UBL4A) to form a trimeric complex that functions in the Guided Entry of Tail (GET)-anchored protein system, found in yeast and mammals (Mariappan et al., 2010; Mock et al., 2015, 2017; Chio et al., 2017; Kuwabara et al., 2015; Krenciute et al., 2013; Shao et al., 2017; Leznicki et al., 2013). For this, nascent polypeptides containing C-terminal, hydrophobic transmembrane domains are bound by the ribosome-associated small glutamine-rich tetratricopeptide repeat-containing alpha (SGTA) and transferred via the BAG6 complex to the transmembrane recognition complex 40 (TRC40) for delivery to the ER membrane (*reviewed in* (Benarroch et al., 2019)). Alternatively, clients transferred to BAG6 itself are committed to degradation through BAG6 association with various PQC effectors. For example, BAG6 has been shown to interact with the proteasomal subunits Rpt4, Rpt6, and Rpn10, suggesting that it can directly feed clients to the proteasome (Hamazaki et al., 2007; Minami et al., 2010; Akahane et al., 2013; Payapilly and High, 2014). In addition, the N-terminal ubiquitin-like (UBL) domain of BAG6 recruits various E3 Ub-ligases, most notably RNF126, that can ubiquitylate and facilitate proteasome-mediated degradation of clients (Hessa et al., 2011; Rodrigo-Brenni et al., 2014; Krysztofinska et al., 2016; Yau et al., 2017; Hu et al., 2020). BAG6 was also shown to interact with ER-associated degradation (ERAD) machinery and participate in the removal of aberrant proteins retrotranslocated from the ER (Wang et al., 2011; Claessen and Ploegh, 2011; Xu et al., 2012, 2013; Payapilly and High, 2014; Hu et al., 2020).

During pathological conditions, the human TAR DNA-Binding Protein 43 (TDP43) protein undergoes proteolytic cleavage at a number of locations giving rise to a variety of proteolytic fragments susceptible to intracellular aggregation (Zhang et al., 2007; Nonaka et al., 2009b; Igaz et al., 2009; Yamashita et al., 2012; Chang et al., 2013; Cohen et al., 2015; Li et al., 2015; Kitamura et al., 2016; Kametani et al., 2016; Rabdano et al., 2017; Kasu et al., 2018; Chhangani et al., 2021). In particular, owing to a C-terminal prion-like domain, C-terminal fragments of TDP43 are the major constituents of proteinaceous aggregates found in cytoplasm of neurons of ALS and FTLD patients (Neumann et al., 2006; Zhang et al., 2007, 2009; Igaz et al., 2009; Nonaka et al., 2009a, 2009b; Budini et al., 2012; Yamashita et al., 2012; Li et al., 2015; Kametani et al., 2016; Kitamura et al., 2016). Such aggregates were shown to be associated with ubiquitin suggesting that defects in their UPS-mediated degradation may play a contributing role (Kasu et al., 2018; Nonaka et al., 2009a; Li et al., 2011; Braak et al., 2010). In previous work, we found that differences in the N-termini of otherwise identical C-terminal fragments can influence their metabolism and aggregation dynamics (Kasu et al., 2018). Of note, we found that degradation by the Arg/N-degron pathway precludes the aggregation of proteolytic fragments bearing N-degrons (N-terminal degradation signals) consisting of a basic (e.g., Arg, Lys, and His) or bulky hydrophobic (e.g., Phe, Lue, Trp, Tyr, and Ile) N-terminal amino acid (Brower et al., 2013; Kasu et al., 2018; Varshavsky, 2011). However, N-degron formation is not a requisite outcome of proteolytic cleavage. As such, not all proteolytic fragments are substrates of the Arg/N-degron pathway. Furthermore, defects in the N-degron pathway, e.g., as a result of age-related decline in activity or exhaustion because of substrate overproduction, allow many substrates to escape degradation by the Arg/N-degron pathway. To determine the fate of proteolytic fragments that escape the N-degron mediated degradation, we inactivated the N-degrons of TDP43^219^ and TDP43^247^, two specific disease-linked fragments of human TDP43 consisting of amino acids 219 - 414 and 247 - 414, respectively. These fragments are 85% identical and differ by an extended hydrophobic N-terminal 28 residue in TDP43^219^ absent in TDP43^247^ (Brower et al., 2013; Kasu et al., 2018). Whereas TDP43^247^ accumulates and forms abundant, large and morphologically distinct aggregates in the absence of the Arg/N-degron pathway, TDP43^219^ forms sparse, tiny aggregates (Kasu et al., 2018). This indicates that an additional PQC mechanism participates in the metabolism of TDP43^219^ and likely discriminates against differences in hydrophobic content. Here, we found that BAG6 prevents protein aggregation by functioning as a sensor of solvent-exposed hydrophobicity in proteolytic fragments. Whereas BAG6 does not recognize full-length TDP43, it binds strongly to TDP43^219^ because of its exposed hydrophobic N-terminus and prevents its intracellular aggregation both by increasing its solubility and by facilitating its RNF126-mediated ubiquitylation. We also provide evidence that BAG6 effects are not limited to fragments of TDP43 but can interact with and solubilize fragments of the amyloid precursor protein. Therefore, BAG6 plays a general role in preventing intracellular aggregation associated with neurodegeneration.

## Results

### BAG6 associates with TDP43^219^ and TDP43^247^

**Figure 1:**
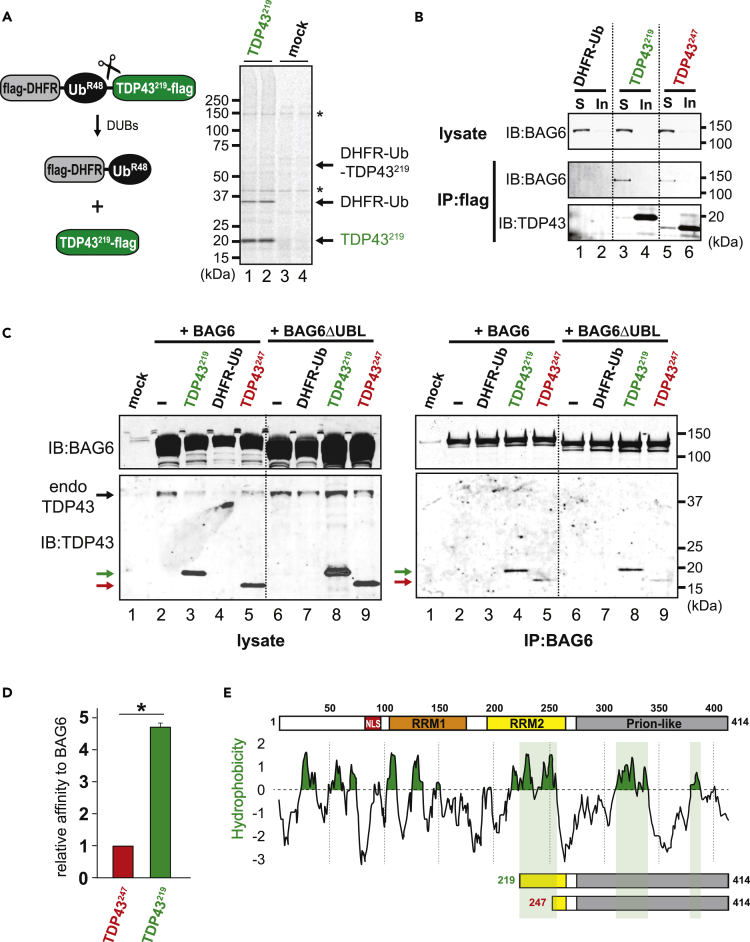
BAG6 interacts with TDP43 proteolytic fragments (A) Proteolytic fragments were expressed in mammalian cells using the ubiquitin reference technique (Varshavsky, 2005). Co-translational cleavage by cellular deubiquitylases (DUBs) yields N-terminally flag-tagged DHFR-Ub^R48^ (which contains a K48R mutation to prevent its participation in polyubiquitin chains) and a test fragment (e.g., TDP43^219^) bearing a specified N-terminal amino acid and a C-terminal FLAG epitope tag. DHFR-Ub^R48^ serves as an internal reference protein. URT-expressed flag-DHFR-Ub-Val-TDP43^219^-flag was labeled with [^35^S] Met, followed by denaturing immunoprecipitation with anti-FLAG, SDS-PAGE, and autoradiography. (B) Exogenously expressed TDP43^219^ and TDP43^247^ were immunoprecipitated (IP) from the detergent-soluble (*S*) and -insoluble (*In*) fractions of HEK293T cells using an anti-FLAG antibody. Endogenous BAG6 was identified in the lysates and IP fractions using an anti-BAG6 antibody. TDP43^219^ and TDP43^247^ were identified in the IP fractions using an anti-TDP43 antibody. (C) Co-IP of DHFR-Ub, TDP43^219^, and TDP43^247^ with wild type BAG6 and BAG6 lacking its N-terminal UBL domain (BAG6ΔUBL) expressed in HEK293T cells. Note that endogenous, full-length TDP43 is not immunoprecipitated by BAG6 or BAG6ΔUBL. *Mock*, mock-transfected. (D) Relative affinity of TDP43^219^ versus TDP43^247^ when co-immunoprecipitated using an anti-BAG6 antibody. Experiments were carried out in triplicate. Error bars indicate standard error of the means (SEM). An independent t-test yielded significant differences between TDP43^219^ and TDP43^247^, t(4) = -22.9, (∗, p < 0.001). (E) Kyte-Doolittle plot showing regions of hydrophobicity (in green) throughout human full-length TDP43. Hydrophobic regions shaded in light green are exposed in C-terminal fragments of TDP43. *NLS*, nuclear localization signal, *RRM*, RNA recognition motif.

To examine proteolytic TDP43 fragments bearing their natural, cleavage-exposed, N-terminus, we use the ubiquitin (Ub)-reference technique (URT) which involves fusing Ub between a downstream test protein and an upstream, long-lived reference protein such as dihydrofolate reductase (DHFR) (Figure 1A). Rapid (co-translational) cleavage by intracellular deubiquitylases (DUBs) after the last residue of Ub enables the initial equimolar expression of a fragment with a specified N-terminal amino acid and an internal reference protein from a single RNA transcript (Varshavsky, 2005). Through immunolabeling, we have been unable to detect full-length URT fusion products, suggesting that DUB cleavage is highly efficient. Nonetheless, inefficient cleavage would lead to confounding results particularly in downstream aggregation studies. Therefore, to confirm efficient and rapid DUB cleavage of URT fusions, we examined their expression using metabolic labeling. For this, we labeled flag-DHFR-Ub-TDP43^219^-flag expressing cells with [^35^S] Met/Cys, blocked translation with cycloheximide, and immediately carried out denaturing anti-FLAG immunoprecipitation to isolate URT products in conditions that prevent further processing (Tansey, 2007). These conditions facilitate highly sensitive detection of newly-formed URT products. Using this approach, we detected both flag-DHFR-Ub and TDP43^219^-flag, which migrate through SDS-PAGE at the expected sizes of ∼33 and ∼21 kDa, respectively. On the other hand, the uncleaved flag-DHFR-Ub-TDP43^219^-flag fusion (calculated molecular mass of 54.6 kDa) was only slightly detected (if at all) above background (Figure 1A, *compare lanes* 1 *and 2 with 3 and 4*). These results indicate that DUB cleavage of URT fusion products is highly efficient and occurs co-translationally.

Previously, we found that TDP43^247^ is degraded exclusively by the Arg/N-degron pathway; whereas TDP43^219^ was degraded even in its absence (Kasu et al., 2018). To identify additional PQC mechanisms that prevent the aggregation of proteolytic fragments, we found that BAG6 interacts with soluble forms of TDP43^219^ and TDP43^247^. To confirm this interaction, we used the URT to express TDP43 fragments bearing N-terminal Val in HEK293T cells treated with the proteasome inhibitor, MG132 to prevent their recognition by the Arg/N-degron pathway and degradation by the proteasome. C-terminally single FLAG-tagged TDP43^219^ and TDP43^247^ were immunoprecipitated from detergent-soluble and -insoluble (urea solubilized) fractions using an anti-FLAG antibody. Although the bulk of TDP43 fragments were detected in the insoluble fractions (consistent with their tendency to aggregate in the absence of their degradation (Brower et al., 2013)), endogenous BAG6 was co-immunoprecipitated with TDP43^247^, and especially with TDP43^219^ but not with DHFR-Ub, in the soluble fractions (Figure 1B).

To further validate the BAG6 interaction with TDP43 fragments, we performed a reciprocal co-IP with an anti-BAG6 antibody from the soluble fractions of cells overexpressing TDP43 fragments and wild type BAG6 or BAG6 lacking its N-terminal UBL domain (BAG6ΔUBL). Despite similar expression levels, BAG6 and BAG6ΔUBL interact with TDP43^219^ and TDP43^247^, albeit less strongly to TDP43^247^ (Figure 1C, *compare IP lanes 4 and 5, and* 8 *and 9*). This result indicates that the N-terminal UBL domain of BAG6 is dispensable for this interaction with proteolytic fragments of TDP43. Densitometry of TDP43 proteolytic fragments interacting with BAG6 revealed a ∼5-fold higher affinity of BAG6 for TDP43^219^ relative to TDP43^247^ (Figure 1D). This is consistent with an extended hydrophobic N-terminus of TDP43^219^ that is not present in TDP43^247^ (Figure 1E). Interestingly, BAG6 did not interact with endogenous full-length TDP43 harboring the same and additional regions of hydrophobicity (Figures 1C and 1E), owing to the absence of their solvent-exposure in the correctly folded protein. Collectively, these results indicate that BAG6 functions as a sensor of proteolytic fragments bearing solvent-exposed hydrophobicity.

### BAG6 solubilizes TDP43 protein fragments

**Figure 2:**
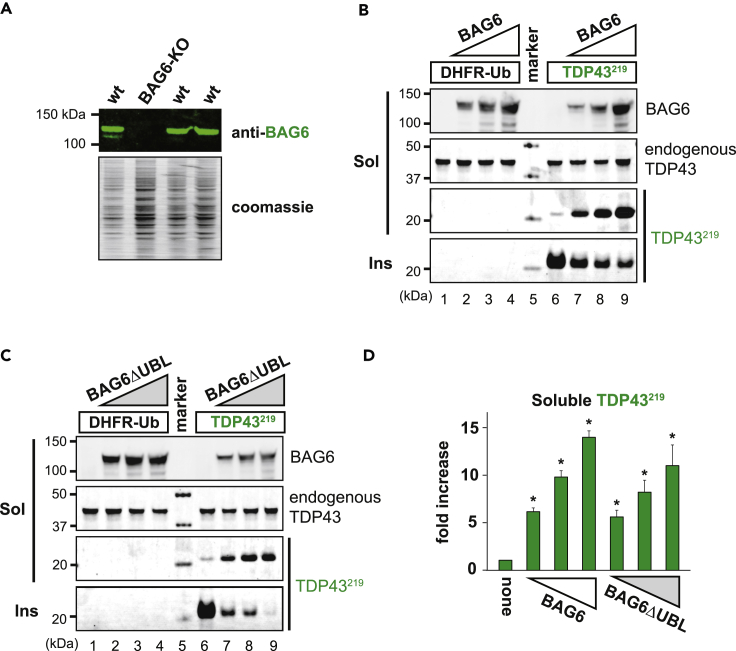
BAG6 solubilizes TDP43^219^ (A) *Upperpanel*, BAG6 is detected by immunoblot using an anti-BAG6 antibody in soluble lysates of wild type HEK293T cells but not clones that have undergone CRISPR-Cas9-mediated *BAG6* ablation (BAG6-KO). *Lower panel*, coomassie stain of the membrane to indicate relative amounts of lysate loaded onto gel. (B) BAG6-KO cells were transfected with TDP43^219^ and increasing amounts of a plasmid encoding wild type BAG6. Cells were lysed and fractionated into detergent-soluble (*Sol*) and -insoluble (*Ins*) fractions. TDP43^219^ and endogenous, full-length TDP43 was detected using a C-terminal anti-TDP43 antibody. DHFR-Ub was used as a control. (C) Same as in B, except using BAG6 lacking its N-terminal UBL domain (BAG6ΔUBL). (D) Relative levels of TDP43^219^ in soluble fractions of BAG6-KO cells as a result of increasing amounts of BAG6 or BAG6ΔUBL. Experiments were carried out in triplicate. A one-way ANOVA demonstrated significant between group differences in soluble TDP43^219^ as a result of increasing concentrations (0, 0.5, 1.0, and 2.0 μg) of BAG6-or BAG6ΔUBL-expressing plasmid (F(6,20) = 14.98, p < 0.01). Fisher LSD post-hoc tests revealed significant differences between samples lacking BAG6 and those containing BAG6 or BAG6ΔUBL. Error bars indicate standard error of the means (SEM). (∗ relative to samples lacking BAG6, p < 0.05).

To ablate BAG6 function in cells, we targeted exon four of the human *BAG6* gene in HEK293T cells using CRISPR-Cas9 and clonally expanded cells lacking BAG6 (BAG6-KO; Figure 2A). To examine TDP43^219^ solubility in the presence and absence of BAG6, we expressed TDP43^219^ (and DHFR-Ub as a control) using the URT (Figure 1A) in BAG6-KO cells in the presence of increasing amounts of plasmid expressing BAG6. In the absence of BAG6, the bulk of TDP43^219^ was detected in the insoluble fraction (Figures 2B and 2C). In contrast, we observed a dose-dependent increase in the levels of soluble TDP43^219^ concomitant with decreased insoluble TDP43^219^ upon titration of exogenous BAG6-expressing plasmids (Figure 2B, *lanes 7–9* and 2D). Notably, BAG6 had no effect on the levels of endogenous TDP43, consistent with its lack of affinity for the full-length correctly folded TDP43. Because the N-terminal UBL domain of BAG6 is dispensable for its interaction with TDP43^219^ (Figure 1C), we asked if it was required for BAG6 effects on TDP43^219^ solubility. BAG6ΔUBL had similar effects on TDP43^219^ solubility as wild type BAG6 (Figures 2C and 2D). These data indicate that TDP43^219^ is largely insoluble in the absence of BAG6 and that BAG6 increases its solubility in a manner that does not require its N-terminal UBL domain.

### BAG6 prevents the oligomerization of TDP43 fragments

**Figure 3:**
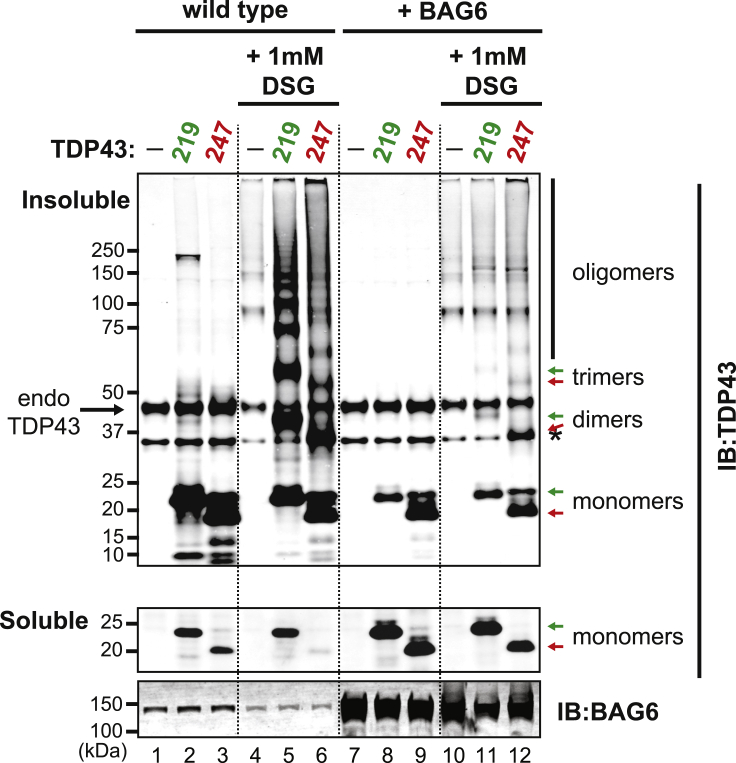
BAG6 prevents the oligomerization of TDP43 proteolytic fragments HEK293T cells were either mock transfected (−) or transfected with plasmids expressing either TDP43^219^ or TDP43^247^ in the presence or absence of exogenously overexpressed BAG6. To detect oligomers, cell pellets were treated with 1 mM disuccinimidyl glutarate (DSG) and lysates were fractionated into detergent-soluble and -insoluble (urea-soluble) factions. TDP43 fragments were detected in the soluble and insoluble fractions by immunoblotting using an anti-TDP43 antibody. Endogenous and exogenous BAG6 was detected by immunoblotting using an anti-BAG6 antibody. Note that dimers of TDP43^247^ overlap with endogenous nonspecific bands denoted by a*sterisks* (*lanes 6 and 12*).

To determine if BAG6 overexpression prevents the formation of TDP43 oligomers and higher-ordered aggregates, we expressed TDP43^219^ and TDP43^247^ in the presence and absence of exogenously overexpressed BAG6 and treated cell pellets with the cross-linking agent, disuccinimidyl glutarate (DSG), to capture and preserve oligomeric species formed within cells. Monomeric and oligomeric species of TDP43 were then detected in lysates by immunoblot using an anti-TDP43 antibody. Consistent with BAG6 interaction with TDP43 fragments and its effects on TDP43^219^ solubilization, the overexpression of BAG6 resulted in higher levels of monomeric TDP43^219^ and TDP43^247^ in the soluble fraction (Figure 3, *compare lanes 2 and 3 to 8 and 9*). Treatment with DSG captured oligomeric species of both TDP43^219^ and TDP43^247^ as detected by a “ladder” of higher molecular weight species in the insoluble fractions (Figure 3, *compare lanes 2 and 3 to 5 and 6*). Strikingly, overexpression of BAG6 resulted in far fewer oligomeric TDP43^219^ and TDP43^247^ species captured in the presence of DSG (Figure 3, *compare lanes 5 and 6 to 11 and 12*). Consistent with a greater affinity to TDP43^219^, the reduction of insoluble oligomeric species in the presence of BAG6 was greater for TDP43^219^ than for TDP43^247^ (Figure 3, *compare lanes 5 and 11 to 6 and 12*). The loss of oligomeric species in the presence of overexpressed BAG6 indicates that BAG6 prevents the self-assembly and oligomerization of neurodegeneration-associated proteolytic fragments of TDP43.

### BAG6 prevents aggregation of TDP43^219^ fragments in cells

**Figure 4:**
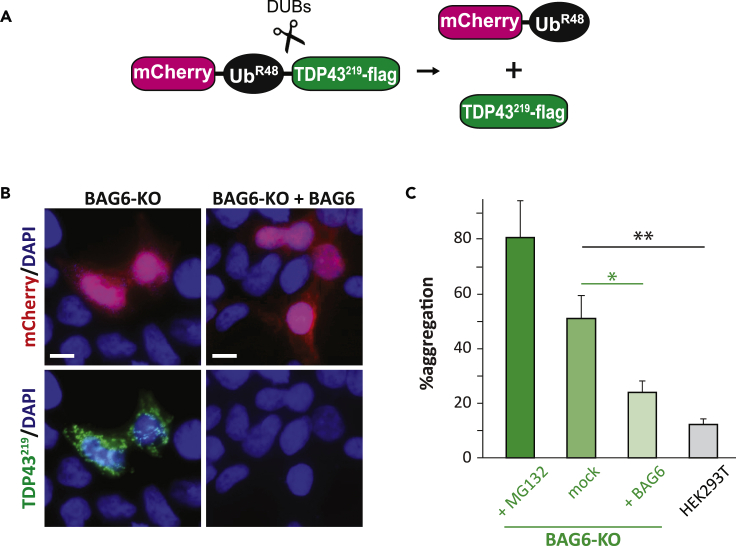
BAG6 prevents intracellular aggregation of TDP43^219^ (A) A modified version of the URT whereby DHFR is replaced by mCherry to identify cells expressing TDP43^219^. (B) Representative images of BAG6-KO cells expressing mCherry-Ub^K48R^-TDP43^219^ in the presence or absence of BAG6. *Upper panels,* transfected cells are detected by mCherry-Ub red fluorescence. *Lower panels,* Aggregates were detected using an anti-FLAG antibody and an Alexa fluor488-conjugated secondary antibody. Bars indicate 10μm. DAPI, 4,6-diaminidino-2-phenylindole. (C) Percentage of mCherry positive cells containing detectable aggregates. Error bars indicate standard errors of the means (SEM). Experiments were carried out in triplicate. At least 600 transfected cells were analyzed for each group. A one-way ANOVA revealed a significant effect of BAG6 knockout on protein aggregation (F(3,13) = 13.661, p = 0.001) with Fisher LSD post hoc tests showing significant group differences. Asterisks represent bars that are significantly different from mock-treated BAG6-KO cells (∗, p = 0.025; ∗∗, p = 0.006).

Previously, we reported that in the absence of degradation by the Arg/N-degron pathway, the majority of TDP43^247^ expressing cells contain cytoplasmic, perinuclear aggregates of various morphologies; whereas cytosolic aggregates were detected in only ∼10% of TDP43^219^ expressing cells (Kasu et al., 2018). Because previous results were obtained in BAG6-containing cells, it is possible that BAG6 prevents significant aggregation of TDP43^219^, especially in light of the current data. To determine if BAG6 prevents the aggregation of TDP43^219^, we compared the expression of TDP43^219^ in HEK293T and BAG6-KO cells using a modified version of the URT where the cDNA encoding DHFR was replaced with cDNA encoding the red-fluorescent mCherry (Figure 4A). Co-translational cleavage of this fusion construct yields a stable mCherry-Ub^R48^ which “marks” transfected cells, and a C-terminally FLAG epitope-tagged TDP43 fragment whose fate can be monitored using indirect immunofluorescence with an anti-FLAG primary antibody and a fluorescein-conjugated secondary antibody (Figure 4B). Consistent with earlier published results, aggregates of TDP43^219^ (quantified as the number of mCherry expressing cells that also contain aggregates) could be detected in only ∼12% of wild type (HEK293T) cells (Figure 4C). As evidence that the UPS is largely responsible for preventing intracellular aggregation of TDP43^219^, aggregates could be detected in ∼80% of treated with the proteasome inhibitor, MG132 (Figure 4C). Remarkably, cytosolic TDP43^219^ aggregates were detected in greater than 50% of BAG6-lacking cells (Figures 4B and 4C). To establish that BAG6 mitigates aggregation, we measured the levels of TDP43^219^ aggregates in BAG6-KO cells following transfection with a BAG6-expressing plasmid. Exogenous expression of BAG6 reduced the levels of TDP43^219^ aggregates in BAG6-KO cells from ∼50% to ∼24% (Figures 4B and 4C). These results indicate that BAG6 prevents the intracellular aggregation of TDP43^219^.

### TDP43^219^ interacts with a BAG6 subcomplex

**Figure 5:**
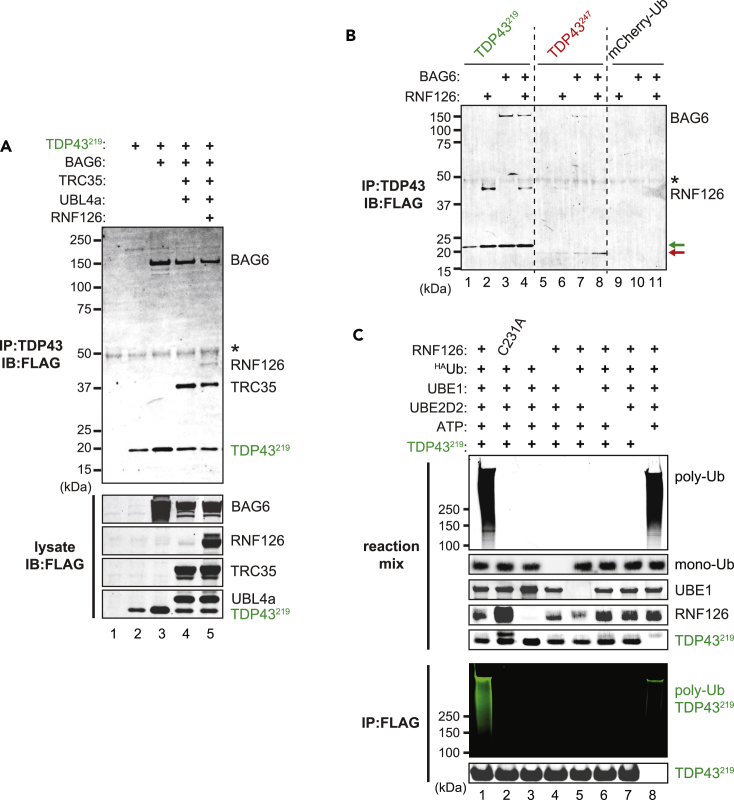
TDP43^219^ interacts BAG6, TRC35, and RNF126 and is associated with RNF126-catalyzed ubiquitylation (A) BAG6-KO cells were transiently transfected with FLAG epitope-tagged TDP43^219^, BAG6, TRC35, UBL4a and RNF126. *Upper panels*, Proteins interacting with TDP43^219^ were detected by IP using anti-TDP43, followed by immunoblot using anti-FLAG. *Asterisk*, antibody heavy chain. *Lower panels*, anti-FLAG immunoblot of lysates. (B) BAG6-KO cells were co-transfected with plasmids expressing TDP43^219^, TDP43^247^, and mCherry-Ub (as a control) and either BAG6, RNF126, or BAG6 and RNF126 together. Proteins associated with TDP43 proteolytic fragments were detected by anti-FLAG immunoblot of an anti-TDP43 co-immunoprecipitation. (C) *In vitro* ubiquitylation reactions containing the indicated components. Total ubiquitylation was detected in reaction mixtures using an anti-ubiquitin antibody. TDP43^219^-specific ubiquitylation was detected by anti-ubiquitin immunoblot of anti-FLAG IP samples. TDP43^219^ was detected using an anti-TDP43 antibody. Additional reaction components were identified through coomassie staining of lysate membrane.

To function in the GET pathway, BAG6 assembles into a trimeric complex with TRC35 and UBL4A (Mariappan et al., 2010; Mock et al., 2015, 2017; Chio et al., 2017; Kuwabara et al., 2015; Krenciute et al., 2013; Shao et al., 2017; Leznicki et al., 2013). To function in PQC, BAG6 also interacts with various E3 Ub-ligases (Hessa et al., 2011; Rodrigo-Brenni et al., 2014; Krysztofinska et al., 2016; Yau et al., 2017; Hu et al., 2020). To determine which, if any, BAG6 partners are associated with quality control of TDP43^219^, we co-expressed FLAG-tagged TDP43^219^, BAG6, TRC35, Ubl4a, and RNF126 in BAG6-KO cells, and carried out co-immunoprecipitation from the soluble fraction using an anti-TDP43 antibody followed by anti-FLAG immunoblot (Figure 5A). TDP43^219^ is associated strongly with BAG6 and TRC35 (Figure 5A, *lane 4*). In addition, the E3 Ub-ligase, RNF126, was co-immunoprecipitated with TDP43^219^ along with BAG6 and TRC35 (Figure 5A, *lane 5*). Interestingly, UBL4a was noticeably absent from the complex regardless of the association of RNF126 (Figure 5A, *lane 4 and 5*). This indicates that UBL4a, which is required for the transfer of TA clients from SGTA to TRC40, is dispensable when BAG6 is bound to clients not destined to the ER.

To determine if TDP43^219^ can interact with RNF126 independently of BAG6, we carried out similar co-immunoprecipitations in BAG6-KO cells in the presence and absence of exogenously added BAG6. Remarkably, both TDP43^219^ and TDP43^247^ (albeit at levels near the detection limit) could associate with RNF126 independently, or along with BAG6 (Figure 5B). A direct interaction between TDP43 fragments and RNF126 was not expected. However, there are reports of BAG6-independent RNF126 ubiquitin-ligase activity (Zhi et al., 2013; Benini et al., 2017). Collectively, these results indicate that TDP43^219^ associates with BAG6 and TRC35 (but not UBL4a), and can associate with the Ub-ligase, RNF126, independently or in a complex with BAG6.

### TDP43^219^ is associated with RNF126 ligase-dependent ubiquitylation

**Figure 6:**
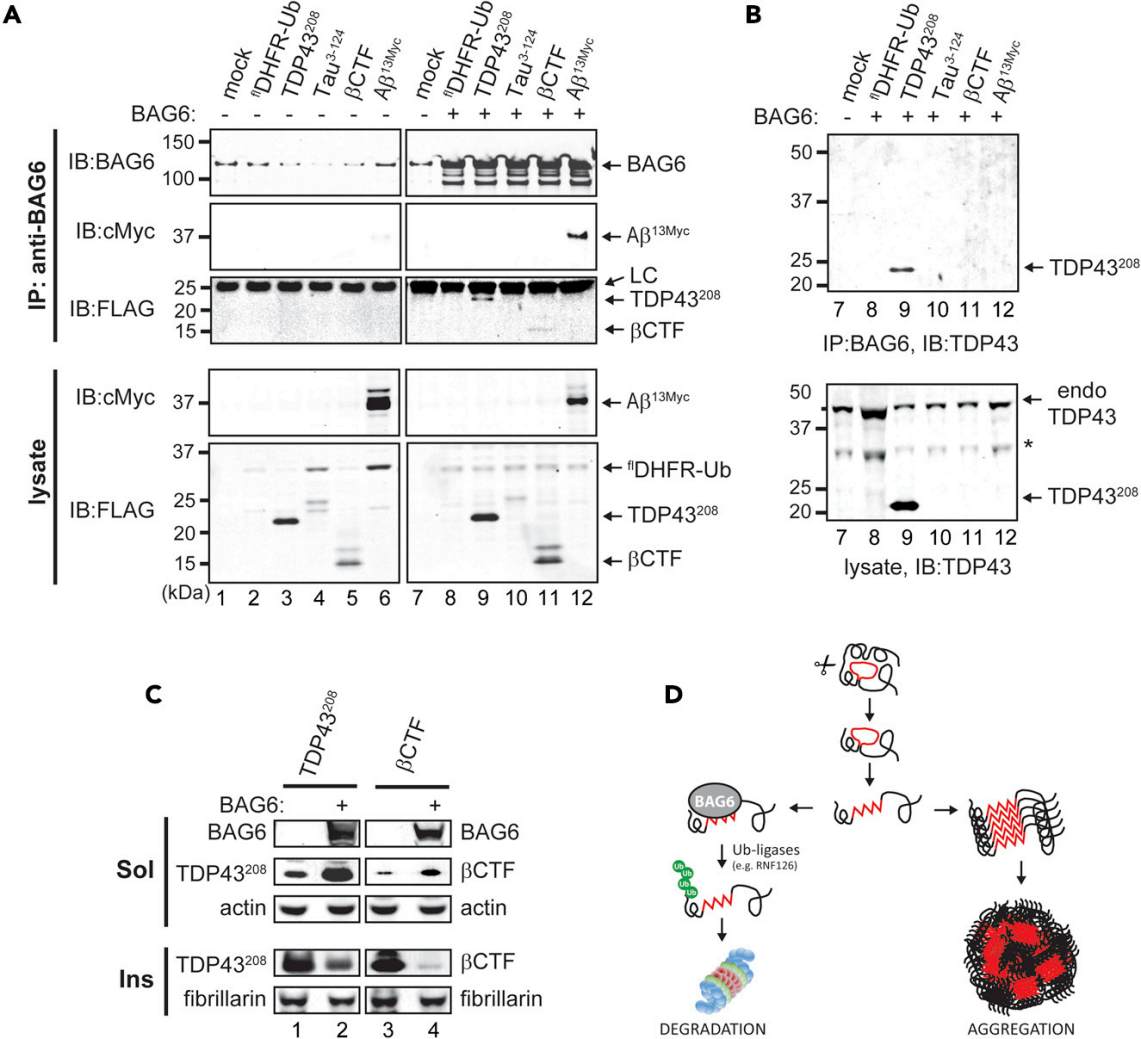
BAG6 has a general role in preventing aggregation of neurodegeneration-associated proteolytic fragments (A) FLAG-tagged DHFR-Ub (as a control), TDP43^208^, Tau, βCTF, and 13myc-tagged Aβ were expressed in the presence of overexpressed BAG6 in HEK293T cells. *Upper panels*, Proteins interacting with BAG6 were detected using an anti-BAG6 co-IP, followed by immunoblot using the indicated antibodies. *Lower panels*, immunoblot of lysates using the indicated antibodies. *Mock*, mock-transfected. *LC*, antibody light chain. (B) IP and lysate fractions from panel A (*lanes 7 through 12*) immunoblotted with an anti-TDP43 antibody. *Asterisk*, endogenous, cross-reacting band. (C and D) BAG6-KO cells expressing either TDP43^208^ or βCTF in the presence or absence of BAG6. Cells were lysed and fractionated into detergent-soluble (*Sol*) and -insoluble (*Ins*) fractions. BAG6, TDP43^208^ and βCTF were detected using an anti-FLAG antibody. Anti-β-actin and anti-fibrillarin was used as loading controls for the soluble and insoluble fractions, respectively (D) Model of BAG6 role in preventing the aggregation of proteolytic fragments. Limited proteolysis (*indicated by scissors*) of various cellular proteins generates misfolded proteolytic fragments with solvent-exposed hydrophobic regions (*red portion of protein*). In the absence of their degradation, these fragments self-associate to form oligomers and insoluble aggregates that are associated with neurodegeneration. Alternatively, hydrophobicity is bound by BAG6 which prevents fragment oligomerization and aggregation. The BAG6 complex can recruit various E3 Ub-ligases—e.g., RNF126—to facilitate client ubiqiutylation and proteasome-mediated degradation.

To determine if RNF126 is capable of ubiquitylating TDP43^219^, we carried out *in vitro* ubiquitylation reactions containing purified HA-tagged Ub, the E1 Ub-activating enzyme (UBE1), the E2 Ub-conjugating enzyme (UBE2), bacterially expressed FLAG-tagged TDP43^219^ and bacterial cell lysates expressing recombinant RNF126, or its catalytically inactive mutant, RNF126^C231A^. Polyubiquitylation, detected by anti-Ub immunoblot, was formed only in reactions that included bacterial lysate containing wild type RNF126 (Figure 5C, *lanes 1 and 8*) and not from those containing non-transformed bacterial lysate or lysate containing RNF126^C231A^ (Figure 5C, *lane 2 and 3*). This indicates that Ub-ligase activity is derived from catalytically active RNF126 and not from another component in the bacterial cell lysate or the reaction mixture. Because polyubiquitylation was also detected in reactions lacking TDP43^219^ (Figure 5C, *lane 8*), its association with TDP43^219^ could not be determined by anti-Ub immunoblot of reaction mixtures. To distinguish TDP43^219^-specific from nonspecific ubiquitylation, we carried out anti-FLAG immunoprecipitation of the *in vitro* reaction mixtures to isolate TDP43^219^ and its associated modifications. Interestingly, polyubiquitylation formed in reaction mixtures lacking TDP43^219^ were not co-immunoprecipitated indicating that it was nonspecific (Figure 5C, *lane 8*). In contrast, significant TDP43^219^-specific polyubiquitylation was detected in immunoprecipitates of reactions containing TDP43^219^ and all other reaction components (Figure 6, *lanes 1*). Collectively, these results indicate that RNF126 can bind TDP43^219^, either directly or can be recruited by BAG6, to catalyze the polyubiquitylation.

### BAG6 interacts with additional neurodegeneration-associated protein fragments

To determine if BAG6 is specific to TDP43^219^ and TDP43^247^ or if it plays a general role in preventing intracellular aggregation of proteolytic fragments with solvent-exposed hydrophobicity, we examined its capacity to interact with TDP43^208^ (amino acids 208–247) and proteolytic fragments linked to AD (Lauritzen et al., 2016; Nunan et al., 2001, 2003). For this, we used the URT (Figure 1A) to co-express BAG6 with FLAG-tagged TDP43^208^, Tau^3-24^, βCTF, or C-terminally 13Myc-tagged Aβ in HEK293T cells. We then carried out co-immunoprecipitation using an anti-BAG6 antibody followed by immunoblot with indicated antibodies to detect association. We were unable to detect expression of Tau^3-24^ of its expected size (∼14 kDa) in soluble fractions; however an interaction between BAG6 and TDP43^208^, βCTF, and Aβ was detected (Figure 6A). Notably, TDP43^208^ is identical to TDP43^219^ except that it contains additional hydrophobic N-terminal amino acids. βCTF and Aβ share a significant region of hydrophobicity that contributes to their propensity to form amyloid deposits. Of note, the highly hydrophilic C-terminal 13Myc-tag, which was added to solubilize the highly amyloidogenic Aβ, did not prevent its interaction with BAG6 (Figure 6A, *lane 12*). We also carried out immunoblot of the lysate and IP fractions using an anti-TDP43 antibody and again found that BAG6 did not interact with endogenous full-length TDP43 protein, presumably because of the lack of exposed hydrophobicity in the correctly folded full-length protein (Figure 6B). To assess the functional significance of these interactions, we examined the levels of TDP43^208^ and βCTF in the soluble and insoluble (urea-solubilized) fractions in the presence and absence of BAG6 expressed in BAG6-KO cells (13Myc-tagged Aβ was not examined as it is not a naturally produced product). Similar to the results obtained with TDP43^219^, the bulk of TDP43^208^ and βCTF was isolated in insoluble fractions in the absence of BAG6. However, co-expression of BAG6 increased the solubility of these polypeptides as seen by their increased partitioning to the soluble fraction (Figure 6C). These results indicate that BAG6 can interact with a variety of aggregation-prone proteins derived from structurally and functionally distinct precursors and can enhance their solubility. Thus, BAG6 plays a general role in preventing the aggregation of neurodegeneration-associated proteolytic fragments.

## Discussion

Previously, we reported that the N-termini of otherwise similar aggregation-prone protein fragments influence their degradation, aggregation dynamics, and potentially the clinical outcomes of neurodegenerative disorders associated with proteinaceous aggregates. For example, TDP43^247^ is degraded exclusively by the Arg/N-degron pathway; but an additional pathway(s) is capable of degrading TDP43^219^ (Kasu et al., 2018). In efforts to identify additional PQC mechanisms that participate in protecting cells from proteinopathies, we found that the molecular chaperone, BAG6, functions as an intracellular sensor of solvent-exposed hydrophobicity in proteolytic fragments. Chaperone-assisted clearance of aggregation-prone proteins and their involvement in the prevention of neurodegenerative diseases is well established and impairment of PQC results in a variety of disease-linked proteinopathies (Ciechanover and Kwon, 2017). We provide a number of independent lines of evidence that BAG6 prevents the intracellular aggregation of neurodegeneration-associated proteolytic fragments. First, using co-immunoprecipitation, we found that BAG6 interacts with specific aggregation-prone fragments of TDP43 (TDP43^208^, TDP43^219^, and TDP43^247^) as well as other proteins associated with neurodegeneration (βCTF and Aβ) (Figure 1 and Figure 6). Second, through biochemical fractionation of CRISPR-Cas9-mediated BAG6-lacking cells, we found that TDP43^219^ is insoluble in the absence of BAG6 but its solubilization is directly correlated to BAG6 levels (Figure 2, Figure 6 and 6). Third, using chemical cross-linking to capture and maintain oligomeric species, we found that BAG6 prevents the self-association and oligomerization of TDP43^219^ and TDP43^247^ (Figure 3). Fourth, using immunocytochemistry, we show that TDP43^219^ forms ∼5-fold more aggregates in BAG6-lacking cells compared to BAG6-containing cells (Figure 4). Lastly, we show that TDP43^219^ can interact with the BAG6-recruited Ub-ligase, RNF126 and is associated with RNF126-catalyzed ubiquitylation (Figure 5). In support of a general function in preventing the aggregation of neurodegeneration-associated fragments, we found that BAG6 also interacts with and solubilizes the amyloidogenic Aβ peptide and the β-secretase generated C-terminal fragment of the amyloid precursor protein, βCTF, involved in AD pathology (Pulina et al., 2020; Checler et al., 2021). Our data support a model (Figure 6D) whereby limited proteolysis during pathological conditions generate proteolytic fragments containing exposed hydrophobicity. A number of specific UPS pathways (e.g., the Arg/N-degron pathway) have been reported to play a role in the clearance of proteolytic fragments associated with neurodegeneration (Hebron et al., 2013; Brower et al., 2013; Uchida et al., 2016; Kasu et al., 2018; Watabe et al., 2020). However those that escape degradation can self-associate, forming higher order oligomers and ultimately insoluble aggregates in attempts to shield exposed hydrophobicity. Alternatively, they are bound by BAG6, maintained in a soluble state to prevent aggregation, and targeted for UPS-mediated degradation through ubiquitylation catalyzed by recruited Ub-ligases, such as RNF126.

Although our data suggests that RNF126 is recruited to TDP43 proteolytic fragments to facilitate their UPS-mediated degradation, it does not preclude the involvement of alternative Ub-ligases as well. BAG6 has been shown to recruit a number of distinct Ub-ligases. For example, the ER-associated Ub-ligase, gp78 is recruited by BAG6 during ERAD of misfolded ER substrates (Wang et al., 2011). In a clever study employing the use of bispecific antibodies to detect K11/K48-linked ubiquitin chains, the BAG6 complex was shown to recruit HUWE1, UBR4, and UBR5 to catalyze the attachment of heterotypic ubiquitin to BAG6-bound substrates (Yau et al., 2017). Interestingly, UBR4 and UBR5 are both Ub-ligases of Arg/N-degron pathway (referred to as “N-recognins” by their ability to recognize N-degrons) (Dougan et al., 2012; Cha-Molstad et al., 2017; Melnykov et al., 2019; Varshavsky, 2019). As such, one possibility is that BAG6 cooperates with the Arg/N-degron pathway in the degradation of proteolytic fragments not only by solubilizing substrates but also through recruitment of UBR4 and UBR5. This is an interesting direction of future study.

BAG6 has been described as a “holdase” or “sortase” chaperone widely studied in the context of post-translational ER-targeting of tail-anchored (TA) proteins bearing hydrophobic C-terminal transmembrane domains. It functions within a trimeric complex containing TRC35 and UBL4a to facilitate client transfer from the upstream co-chaperone, SGTA to the downstream cytosolic ATPase, TRC40 (Mariappan et al., 2010; Mock et al., 2015, 2017; Chio et al., 2017; Kuwabara et al., 2015; Krenciute et al., 2013; Shao et al., 2017; Leznicki et al., 2013). The BAG6 complex also participates in PQC by recruiting Ub-ligases to facilitate client ubiquitylation (Hessa et al., 2011; Rodrigo-Brenni et al., 2014; Krysztofinska et al., 2016; Yau et al., 2017; Hu et al., 2020). Although it does not preclude ubiquitylation by alternative Ub-ligases, we found that RNF126 is recruited to the TDP43^219^-bound BAG6 complex and catalyzes TDP43^219^-associated polyubiquitylation. Interestingly, the TDP43^219^-associated BAG6 complex lacked UBL4a. The reason for UBL4a exclusion is unclear. However there is precedence for BAG6 functions in the absence of UBL4a (Thress et al., 1998; Krenciute et al., 2013). An interesting possibility is that the trimeric BAG6 complex (BAG6/TRC35/UBL4a) undergoes remodeling to accommodate the triage needs of specific client proteins. To maintain proteostasis, the BAG6 complex drives either biosynthetic or PQC agendas determined largely by client dissociation from SGTA and capture by BAG6 (Shao et al., 2017). However, SGTA competes for clients and antagonizes BAG6-mediated degradation by promoting de-ubiquitylation (Leznicki and High, 2012; Wunderley et al., 2014). Structural studies have shown that RNF126 and SGTA compete for the N-terminal UBL domain of BAG6 (Krysztofinska et al., 2016). However, the UBL domain of UBL4a also competes for SGTA binding (Leznicki et al., 2013). As such, BAG6 commitment to PQC could be accomplished by eliminating SGTA antagonizing effects through the recruitment of Ub-ligases and disengagement with UBL4a. This may explain why TDP43^219^-bound BAG6 is not associated with UBL4a (Figure 5A). Further studies are needed to confirm this conjecture.

Although TDP43^219^ and TDP43^247^ are ∼85% identical, TDP43^219^ contains an additional N-terminal 28-amino acids that contribute a significant hydrophobic “handle” not included in TDP43^247^. Consistent with an exposed hydrophobicity sensing function, BAG6 interacts with ∼5-fold affinity for TDP43^219^ than for TDP43^247^ (Figure 1D). This suggests that BAG6 has a graded response in cellular protection as it has the highest affinity for the most aggregation-prone fragments. As such, BAG6 may prioritize removal of the most toxic proteins first.

Although many chaperones are upregulated during stress, BAG6 does not appear to be upregulated in response to the expression of TDP43 fragments (Figure 3, *lanes 1 to 3*). As such, the overproduction of toxic proteolytic fragments may not only exhaust PQC functions but also compete with TA proteins otherwise destined for the ER. Interestingly, a recent study showed that SGTA, which senses hydrophobic transmembrane domains in TA proteins also associates with intracellular aggregates associated with neurodegenerative diseases (Kubota et al., 2021). Consequently, disruption of the GET pathway could be involved in the etiology of neurodegeneration.

In sum, we show that the molecular chaperone, BAG6, plays a role in preventing the intracellular aggregation of proteolytic fragments bearing solvent-exposed hydrophobicity by maintaining their solubility and preventing their self-association. In addition, BAG6 facilitates their ubiquitylation and subsequent degradation by recruiting Ub-ligases such as RNF126. Therefore, BAG6 plays a protective role by preventing intracellular aggregation associated with neurodegeneration.

### Limitations of the study

BAG6 is part of the heterotrimeric complex that dynamically interacts with other proteins involved in protein targeting and UPS-mediated degradation. Thus, interpretation of specific downstream effects of BAG6 (e.g., degradation effects) is difficult using overexpression experiments that disrupt subunit stoichiometry. Our data reveal that RNF126 is capable of interacting with and ubiquitylating TDP43^219^. However, as only one of multiple Ub-ligases recruited by BAG6 to facilitate UPS-mediated degradation, our work does not preclude the recruitment of alternative Ub-ligases. In addition, BAG6 may also play a redundant role in protein quality control. For example, chaperones of the Hsp70 family and Hsp90 also interact with the Ub-ligase CHIP (carboxy-terminus of Hsc70 interacting protein) to triage proteins with small hydrophobic regions (Connell et al., 2001).

## STAR★Methods

### Key resources table

**Table:** 

REAGENT or RESOURCE	SOURCE	IDENTIFIER
**Antibodies**		

Rabbit polyclonal anti-TDP43 (C-terminal)	Proteintech	Cat#12892-1-AP; RRID: AB_2200505
Mouse monoclonal anti-BAG6 (D-1)	Santa Cruz Biotech	Cat# sc-365928; RRID: AB_10920223
Mouse monoclonal anti-Fibrillarin (G-4)	Santa Cruz Biotech	Cat# sc-166021; RRID: AB_2105797
Mouse monoclonal anti-β-Actin (C4)	Santa Cruz Biotech	Cat# sc-47778; RRID: AB_2714189
Mouse monoclonal anti-FLAG M2	Sigma-Aldrich	Cat#F1804; RRID: AB_262044
Mouse monoclonal anti-FLAG M2 magnetic beads	Sigma-Aldrich	Cat#M8823; RRID: AB_2637089
Mouse monoclonal anti-c-Myc (clone 9E10)	Sigma-Aldrich	Cat#M5546; RRID: AB_260581
Goat anti-mouse IgG (H+L)-AlexaFluor488	Thermo Scientific	Cat#A-11001; RRID: AB_2534069
Goat anti-mouse IgG DyLight 680	Thermo Scientific	Cat#35519, RRID: AB_1965956
Goat anti-mouse IgG DyLight 800	Thermo Scientific	Cat#SA5-10176; RRID: AB_2556756
Goat anti-rabbit IgG DyLight 680	Thermo Scientific	Cat#35569; RRID: AB_1965957
Goat anti-rabbit IgG DyLight 800	Thermo Scientific	Cat#SA5-10036; RRID: AB_2556616

**Bacterial and virus strains**		

BL21(DE3) Chemically Competent Cells	Sigma-Aldrich	Cat#CMC0014
Chemicals, peptides, and recombinant proteins		
MG132	Caymen Chemical	Cat#10012628
TRAN^35^S-label	MP Biomedicals	Cat#51009
Di(N-succinimidyl) glutarate	Sigma-Aldrich	Cat#80424
Recombinant human UbcH5b/UBE2D2 protein	R&D systems	Cat#E2-622-100
Recombinant Yeast GDT-Ubiquitin activating enzyme	R&D systems	Cat#E-300-050
Recombinant RNF126 protein	Abnova	Cat#H00055658-P01

**Experimental models: Cell lines**		

Human embryonic kidney (HEK)-293T cells	ATCC	Cat#CRL-3216
BAG6-knockout HEK293T cells	This study	N/A

**Oligonucleotides**		

CB521F: CACCGGGGATCCCCCCCTGGTACT	This study	N/A
CB522R: AAACAGTACCAGGGGGGGATCCCC	This study	N/A
CB532F: GATTGGTACCGGATC CACCATGGTGAGCAAGGGCG	This study	N/A
CB524R: GATTGATATCGAATTCT CACTTGTCATCGTCGTCCT	This study	N/A
CB527F: GATTCCATGGATGTCTTCATCCCCAAG	This study	N/A
CB529R: GATCCATATGTCAGTGATGATGATGA TGATGCATCTTGTCATCGTCGTCCTTGT	This study	N/A
MA012F: GATCTCTAGACTAAGGA TCATCAGCAAAGGCCCGC	This study	N/A
MA013R: GATCCTCGAGCCACCA TGGAGCCTAATGATAGTACCAGTACC	This study	N/A

**Recombinant DNA**		

pcDNA3.0-Neo: Amp^R^; Neo^R^; Expression vector for cloning your gene of interest	Invitrogen	
pET-16b: Amp^R^; Bacterial vector for expressing 10xHis-tagged proteins.	Novagen (EMD Millipore)	
pX330-U6-Chimeric_BB-CBh-hSpCas9: Amp^R^; Mammalian expression plasmid encoding a human codon-optimized SpCas9 and a chimeric guide RNA	Cong et al., 2013	Addgene Cat#42230
pCB264: Amp^R^; Neo^R^; pcDNA3.0-based plasmid encoding ^f^DHFR-Ub^K48R^-Val-Aβ(11-42)^13myc^ under the control of T7 or CMV promoter	Brower et al., 2013	N/A
pCB323: Amp^R^; Neo^R^; pcDNA3.0-based plasmid encoding ^f^DHFR-Ub^K48R^-Met^208^-TDP43^f^ under the control of T7 or CMV promoter	Brower et al., 2013	N/A
pCB328: Amp^R^; Neo^R^; pcDNA3.0-based plasmid encoding ^f^DHFR-Ub^K48R^-Val^219^-TDP43^f^ under the control of T7 or CMV promoter	Brower et al., 2013	N/A
pCB332: Amp^R^; Neo^R^; pcDNA3.0-based plasmid encoding ^f^DHFR-Ub^K48R^-Val^247^-TDP43^f^ under the control of T7 or CMV promoter	Brower et al., 2013	N/A
pCB385: Amp^R^; pcDNA3.0-based plasmid encoding ^f^DHFR-Ub^K48R^-Val-Tau^3-124^ under the control of T7 or CMV promoter	Brower et al., 2013	N/A
pCB396: Amp^R^; Neo^R^; pcDNA3.0-based plasmid encoding ^f^DHFR-Ub^K48R^-Val-CTFβ^f^ under the control of T7 or CMV promoter	Brower et al., 2013	N/A
pCB399: Amp^R^; pGL3-Control-based plasmid encoding mCherry-Ub^K48R^-Val^219^-TDP43^f^ under the control of SV40 promoter	Kasu. et al., 2018	N/A
pYK27: Amp^R^; Neo^R^; pcDNA3.0-based plasmid encoding ^f^DHFR-Ub^K48R^ under the control of T7 or CMV promoter	Kasu. et al., 2018	N/A
pRK5-FLAG-Bag6: Amp^R^; Neo^R^; pRK5-based plasmid encoding ^f^BAG6 under the control of CMV promoter	Liu et al., 2014	Addgene Cat#61836
pRK5-FLAG-Bag6ΔUBL: Amp^R^; Neo^R^; pRK5-based plasmid encoding ^f^BAG6ΔUBL under the control of CMV promoter	Liu et al., 2014	Addgene Cat#61837
pAAV-Ef1a-Cre: Amp^R^; AAV-based plasmid encoding Cre recombinase under the control of EF1α promoter	Fenno et al., 2014	Addgene Cat#55636
p571: Kan^R^; Neo^R^, pCMV6-Entry-based plasmid encoding ^myc-f^TRC35 under the control of CMV promoter	Origene	Cat#RC200220
p572: Kan^R^; Neo^R^, pCMV6-Entry-based plasmid encoding ^myc-f^UBL4A under the control of CMV promoter	Origene	Cat#RC208121
p573: Kan^R^; Neo^R^, pCMV6-Entry-based plasmid encoding ^myc-f^RNF126 under the control of CMV promoter	Origene	Cat#RC200161
GST-RNF126: Amp^R^; pGEX-4T3 based plasmid for bacterial expression of ^GST^RNF126	Smith et al., 2013	Addgene Cat#138643
GST-RNF126^C231A^: Amp^R^; pGEX-4T3 based plasmid for bacterial expression of ^GST^RNF126^C231A^	Smith et al., 2013	Addgene Cat#138644
pYK33: Amp^R^; pET16b-based plasmid for bacterial expression of Met^219^-TDP43^f-6XHis^	This study	N/A
pJH551: Amp^R^; pUC Ori vector (pX330) based plasmid encoding a human codon-optimized SpCas9 and a chimeric guide RNA for human BAG6	This study	N/A
pCB557: Amp^R^; AAV-based plasmid encoding mCherry-Ub^K48R^-Val^219^-TDP43^f^ under the control of EF1α	This study	N/A
pCB563: Amp^R^; NeoR, pcDNA3.0- based plasmid encoding untagged BAG6 under the control of CMV promoter	This study	N/A

### Resource availability

#### Lead contact

Further information and requests for resources and reagents should be directed to and will be fulfilled by the lead contact, Christopher Brower (cbrower@twu.edu).

#### Materials availability

Plasmids and cell lines generated in this study are available on request.

### Experimental model and subject details

Assays performed in this study utilized Human embryonic kidney (HEK)-293T cells originally received and authenticated from American Type Culture Collection (ATCC). BAG6-KO cells were derived from HEK293T cells.

#### Cell culture and transfection

Human embryonic kidney (HEK)-293T cells were cultured in Dulbecco Modified Eagle Medium (DMEM) (Corning Cellgro) supplemented with 10% fetal bovine serum (Gemini Bio-products). The media also contained 20 mM glutamine, 100 U/ml penicillin and 0.1 mg/mL streptomycin (Fischer Bioreagents). BioT (Bioland Scientific) was used to transfect plasmids as per manufacturer’s protocol.

#### Generation of BAG6-lacking HEK293T cells

HEK293T lacking BAG6 were generated using the CRISPR-Cas9 system (Cong et al., 2013). Briefly, pJH551, a plasmid encoding a human codon-optimized *Sp*Cas9 and a chimeric guide RNA targeting the exon 4 of human *BAG6* gene, was constructed by the ligation of a double strand oligomer (made by the denaturation and renaturation of CB521F and CB522R) into BbsI digested pX330 (pX330-U6-Chimeric_BB-CBh-hSpCas9 was a gift from Feng Zhang; Addgene plasmid # 42230). Cells were transfected with pJH551 and individual clones were selected and screened for loss of the BAG6 protein by immunoblot (Figure 2A).

#### Miscellaneous reagents

MG132 was from Caymen Chemical. Di(N-succinimidyl) glutarate (DSG) was from Sigma-Aldrich. Anti-C-terminal TDP43 (12892-1-AP) was from Proteintech. Anti-BAG6 (sc-365928), anti-fibrillarin (sc-166021), and anti β-Actin (sc-47778) were from Santa Cruz Biotechnology. Anti-FLAG M2 (F1804), anti-FLAG M2 Magnetic Beads (M8823), and anti-Myc (M5546) was from Sigma-Aldrich. Secondary antibodies: anti-mouse IgG (H + L)-AlexaFluor488 (A-11001), anti-mouse IgG DyLight 680 (35519), anti-mouse IgG DyLight 800 (SA5-10176), anti-rabbit IgG DyLight 680 (35569), and anti-rabbit IgG DyLight 800 (SA5-10036) were from Thermo Scientific.

### Methods details

Plasmids: PRK5-FLAG-Bag6 and PRK5-FLAG-Bag6ΔUBL were gifts from Yihong Ye (Addgene plasmid # 61836 and 61837, respectively) (Liu et al., 2014). GST-RNF126 and GST-RNF126 C231A were gifts from Jane McGlade (Addgene plasmid # 138643 and 138644, respectively) (Smith et al., 2013). To generate pCB557, DNA encoding mCherry-Ub-Val 219-TDP43^f^ was amplified from pCB399 using primers CB523F and CB524R and used to replace the Cre cDNA in pAAV-Ef1a-Cre after digestion with KpnI/EcoR1. (pAAV-EF1a-Cre was a gift from Karl Deisseroth; Addgene plasmid # 55636) (Fenno et al., 2014). To generate pCB563, DNA encoding BAG6 was amplified from pRK5-FLAG-Bag6 with primers MA012R and MA013F, and inserted into pcDNA3.0 after digestion with xhoI/xbaI. To generate pYK33, Met^219^-TDP43^f^ was amplified from pCB323 using primers CB527F and CB529R and inserted into pET16b vector backbone after digestion with NcoI/NdeI.

#### Protein aggregation

Wild type and BAG6-lacking HEK293T cells were transfected at ∼70% confluency in chamber slides with plasmids expressing TDP43^219^ (pCB399) in the presence and absence of plasmids expressing untagged BAG6 (pCB563). After expression for forty-eight hours, 4% formaldehyde was used to fix the cells by treating for 10 min at room temperature, followed by three, 10 min washes at room temperature in phosphate buffered saline (PBS), and treatment with 0.5% Triton X-100 in PBS for 10 min at room temperature to permeabilize the fixed cells. After permeabilization, the slides are washed again thrice with PBS and processed for indirect immunocytochemistry. For blocking, the slides were incubated in 10% goat serum (Thermofisher Scientific) at 37°C for 1 h. To detect C-terminally FLAG tagged TDP43^219^, slides were incubated with an anti-FLAG antibody (1:1000 dilution) in “wash buffer” (PBS containing 5% goat serum and 0.1% Tween 20) at 37°C for 2 h, followed by three, 10 min washes with wash buffer at room temperature. Slides were then incubated with goat anti-mouse Alexa fluor488 secondary antibody at 1:500 dilution in wash buffer at 37°C for 1 h. Finally, slides were washed again three times with wash buffer at room temperature and once in PBS before mounting in 4′,6-diamidino-2-phenylindole (DAPI)-containing Vectashield H-1200 mounting medium (Vector Laboratories). Fluorescence for mCherry, DAPI, and Alexa fluor488-conjugated secondary antibody was examined using a Nikon A1 Confocal Microscope. Experiments were done in triplicates. Transfected cells were counted in at least ten randomly selected fields. At least 600 transfected cells were analyzed in total for each group. Statistical analysis included a one-way analysis of variance (ANOVA) test followed by a Fisher LSD post-hoc test. p < 0.05 was required for significance.

#### Lysate preparation, immunoprecipitation and immunoblotting

Cultured cells were trypsinized, washed in PBS, and lysed by freeze/thawing in “tissue lysis buffer” (TLB) (50 mM HEPES (pH 7.5), 10% glycerol, 0.05% NP-40, 150 mM NaCl, 1mM dithiothreitol (DTT) and 1 mM phenylmethylsulfonyl fluoride (PMSF) containing Pierce protease-inhibitor mixture). To obtain soluble fractions, lysates were centrifuged at 13,000 rpm for 15 min at 4°C and the supernatants were collected. To obtain insoluble fractions, pellets from the first spin were washed twice in TLB, resuspended by sonication in 8M urea buffer (8 M urea, 50 mM Tris (pH 8.0), 1 mM DTT and 1 mM PMSF), and centrifuged at 13,000 rpm for an additional 10 min to recover the final supernatant. Protein concentrations were determined using the BioRad Protein Assay reagent (BioRad) and normalized before immunoprecipitation or immunoblotting. For immunoprecipitation, 10 μL of Protein G magnetic beads slurry (BioRad) were washed three times with TLB and then incubated with 0.5 μg anti-FLAG-M2 antibody, anti-TDP43 C-terminal antibody, or anti-BAG6 antibody (per sample) for 30 min at 4°C. The antibody-bead mixtures were added to normalized protein lysates (200μg) and rotated for an additional 2 h at 4°C. Then, beads were magnetically separated from the lysate and the “non-bound” proteins were collected. Beads were washed three times with TLB at 4⁰C (with rotation) and ultimately resuspended in 2× SDS-PAGE protein loading buffer (PLB). For immunoblotting, PLB containing samples were heated at 95°C for 5 min and separated on a 4-12% gradient NuPage Bis-Tris gels (Invitrogen). The samples were then transferred onto methanol activated PVDF membrane (BioRad), in Towbin buffer (25 mM Tris, 192 mM glycine and 20% methanol) overnight at 30V (constant voltage) at 4⁰C. Membranes were incubated in blocking buffer (5% milk in PBS containing 0.1% Tween 20) at room temperature for 1 h, then incubated with the indicated primary antibodies at 1:1000 dilution (made in blocking buffer) either overnight at 4°C or for 3 h at room temperature. Membranes were then washed three times for 10 min in wash buffer, then incubated with secondary antibodies (1:7000 dilution) for 1 h at room temperature. Thereafter, blots were washed three times with wash buffer, twice in PBS, and developed using Licor Oddysey CLx system or a BioRad ChemiDoc MP system.

#### Metabolic labeling

HEK293T cells (∼75% confluent) were transfected with 0.8 μg of pCB328 per 35-mm well using BioT according to the manufacturer's protocol. At 24 h post-transfection, cells were pulse labeled for 10 min with 0.1 mCi/mL ^35^S-labeled methionine (MP Biomedicals) in DMEM lacking Met and Cys. Labeling was quenched by the addition of 0.1 mg/mL cycloheximide (VWR) in complete DMEM containing Met and Cys. Denaturing immunoprecipitation was then carried out according to the Tansey protocol (Tansey, 2007). Briefly, cells were immediately lysed by rapid scraping in 150 μL of TSD buffer (50 mM Tris-HCl [pH 7.4], 1% SDS, 5 mM DTT) and snap-freezing in liquid nitrogen. Samples were then heated at 95°C for 10 min and diluted with 10 volumes of TNN buffer (0.5% NP-40, 0.25 M NaCl, 5 mM EDTA, 50 mM Tris-HCl [pH 7.4]) containing the complete protease inhibitor mixture (Roche). Samples were then immunoprecipitated by the addition of 5 μl of anti-FLAG-M2 magnetic beads (Sigma) and incubation with rocking at 4°C for 3 h. Immunoprecipitated proteins were then washed 3 times in TNN buffer and once in phosphate-buffered saline, followed by resuspension in 20 μl of SDS sample buffer, heating at 95°C for 10 min, 4 to 15% SDS-PAGE, and autoradiography.

#### Protein cross linking

HEK293T cells were transfected with plasmids expressing DHFR-Ub^K48R^ (pYK27), or TDP43^219^ (pCB328) and TDP43^247^ (pCB332) bearing N-terminal Val from the *P*_*CMV*_ promoter. Forty-eight hours post-transfection, cells were resuspended in 1mM disuccinimidyl glutarate containing phosphate buffered saline and incubated at room temperature with shaking for 30mins. Then, 20mM Tris (pH 8) was added to quench the reaction and incubated at room temperature with shaking for 15mins. The cells were then centrifuged at 1000 x rpm for 3 mins and the cell pellet was resuspended in chilled TLB for protein extraction as described above.

#### Expression and purification of recombinant Met^219^-TDP43^f−6xHis^

pYK33 was transformed into BL21 (DE3) bacterial strain. One liter of LB medium culture of BL21 (DE3) bacteria expressing pYK33 was induced for plasmid expression with 0.25mM isopropyl-β-D-thiogalactoside at 0.6AU, and then grown for 6 h with shaking at 30°C. Then, cells were harvested by centrifugation at 2000 × g for 10 mins at 4°C. The pellet was resuspended in bacterial lysis buffer (20mM Tis (pH 8), 300mM NaCl, 10mM imidazole (pH 8), 20m,M β-mercaptoethanol, 1mg/ml lysozyme, 30% v/v glycerol, and protease inhibitor cocktail (Sigma)) and flash frozen in liquid nitrogen. After thawing slowly on ice, crude lysate was centrifuged at 100,000 × g for 30 mins at 4°C. Because recombinant Met^219^-TDP43^f−6xHis^ localized to inclusion bodies, the pellet was resuspended and homogenized in inclusion body solubilization (IBS)-10 buffer (40mM Tris (pH 8), 6M guanidine hydrochloride, 0.5M KCl, 10mM imidazole (pH 8), and 0.5mM PMSF). For purification of recombinant protein, the solution was clarified by centrifugation at 100,000 × g for 35mins. The supernatant was applied to 0.5mL resin volume Nickel-column pretreated with IBS buffer ((40mM Tris (pH 8), 6M guanidine hydrochloride, 0.5M KCl, and 0.5mM PMSF). The columns were then washed three times with IBS-40 buffer (IBS buffer containing 40mM imidazole) and then eluted in IBS-300 buffer (IBS buffer containing 300mM imidazole). The presence of recombinant TDP43 protein fragment in eluate was detected by immunoblotting with anti-TDP43 antibody.

#### *In vitro* ubiquitylation reaction

Ubiquitylation reactions were adapted from those described in Hu et al., (2020) (Hu et al., 2020). Briefly, ubiquitylation reactions contained ubiquitylation buffer (30 mM HEPES, pH 7.5, 50 mM NaCl, 2.5 mM MgCl_2_, and 0.25 mM DTT), HA-Ubiquitin (10μM), GST-Ube1 (0.25μM), UbcH5b (0.5μM), ATP (2μM), Met^219^-TDP43^f−6xHis^ (500ng) and clarified (13,000rpm for 15 min at 4°C) BL21 (DE3) lysates expressing wild type RNF126 (transformed with GST-RNF126), RNF126^C231A^ (transformed with GST-RNF126^C231A^), or non-transformed BL21 (DE3). The reaction was incubated at 25°C for 1 h, then stopped by freezing in liquid nitrogen. To detect polyubiquitylation associated with TDP43 protein fragments, anti-FLAG antibody was used to immunoprecipitate Met^219^-TDP43^f−6xHis^, followed by immunoblotting with anti-ubiquitin antibody.

### Quantification and statistical analysis

For quantifying intracellular aggregation of TDP43^219^ (Figure 4C), aggregates were detected by immunocytochemistry using an antibody to the C-terminal flag epitope of TDP43^219^ and an Alexa 488-conjugated secondary antibody. Aggregates were scored as the number of mCherry-expressing cells that also display detectable TDP43^219^ aggregates. mCherry positive cells in at least ten randomly selected fields were examined. Experiments were carried out in triplicate. At least 600 transfected cells were analyzed per group. A one-way ANOVA revealed a significant effect of BAG6 knockout on protein aggregation (F(3,13) = 13.661, p = 0.001) with Fisher LSD post-hoc tests showing significant group differences. To determine the relative affinity of BAG6 for TDP43^219^ versus TDP43^247^ (Figure 1D), densitometry of TDP43^219^ and TDP43^247^ co-immunoprecipitated from equal amounts of BAG6 was carried out using ImageJ from three independent experiments. An independent t-test yielded significant differences between TDP43^219^ and TDP43^247^ (t(4) = -22.9, p < 0.001). To determine relative levels of TDP43^219^ in soluble fractions of BAG6-KO cells as a result of increasing amounts of BAG6 or BAG6ΔUBL (Figure 2D), densitometry of soluble TDP43^219^ was carried out using ImageJ and levels obtained in the presence of BAG6 were compared to the levels obtained in the absence of BAG6. A one-way ANOVA demonstrated significant between group differences in soluble TDP43^219^ as a result of increasing concentrations (0, 0.5, 1.0, 2.0 μg) of BAG6-or BAG6ΔUBL-expressing plasmid (F(6,20) = 14.98, p < 0.01). Fisher LSD post-hoc tests revealed significant differences between samples lacking BAG6 and those containing BAG6-or BAG6ΔUBL-expressing plasmid. Experiments were done in triplicate. Data analysis was carried out using the SSPS Statistics 25 software (IBM Corporation). p ≤ 0.05 was required for statistical significance.

## Data Availability

• All immunoblotting and microscopy data reported in this paper will be shared by the lead contact upon request. • This paper does not report original code. We specify tools used in the quantification and statisctical analysis section. • Any additional information required to reanalyze the data reported in this paper is available from the lead contact upon request.
